# Fast virtual histology using X-ray in-line phase tomography: application to the 3D anatomy of maize developing seeds

**DOI:** 10.1186/s13007-015-0098-y

**Published:** 2015-12-18

**Authors:** David Rousseau, Thomas Widiez, Sylvaine Di Tommaso, Hugo Rositi, Jerome Adrien, Eric Maire, Max Langer, Cécile Olivier, Françoise Peyrin, Peter Rogowsky

**Affiliations:** Laboratoire CREATIS, Université de Lyon, CNRS, UMR5220, INSERM, U1044, Université Lyon 1 INSA-Lyon, Villeurbanne, France; Unite Reproduction et Developpement des Plantes, INRA, UMR 879, CNRS, UMR 5667, Université Lyon 1, École Normale Supérieure UMR20, 69364 Lyon, France; MATEIS, UMR CNRS 5510, Université Lyon 1, INSA-Lyon, 69621 Lyon, France

**Keywords:** X ray in-line phase tomography, Image segmentation, Virtual histology, plant development, maize seeds

## Abstract

**Background:**

Despite increasing demand, imaging the internal structure of plant organs or tissues without the use of transgenic lines expressing fluorescent proteins remains a challenge. Techniques such as magnetic resonance imaging, optical projection tomography or X-ray absorption tomography have been used with various success, depending on the size and physical properties of the biological material.

**Results:**

X-ray in-line phase tomography was applied for the imaging of internal structures of maize seeds at early stages of development, when the cells are metabolically fully active and water is the main cell content. This 3D imaging technique with histology-like spatial resolution is demonstrated to reveal the anatomy of seed compartments with unequalled contrast by comparison with X-ray absorption tomography. An associated image processing pipeline allowed to quantitatively segment in 3D the four compartments of the seed (embryo, endosperm, nucellus and pericarp) from 7 to 21 days after pollination.

**Conclusion:**

This work constitutes an innovative quantitative use of X-ray in-line phase tomography as a non-destructive fast method to perform virtual histology and extends the developmental stages accessible by this technique which had previously been applied in seed biology to more mature samples.

**Electronic supplementary material:**

The online version of this article (doi:10.1186/s13007-015-0098-y) contains supplementary material, which is available to authorized users.

## Background

Plant developmental biology and plant functional genetics have been strongly impacted by novel imaging techniques allowing to monitor in a non-destructive manner developmental processes or to compare wild-type and mutant structures. The global analysis of morphological, genetic and biochemical information not only deepens our understanding of developmental processes, it is also the basis for modelling (systems biology) and ultimately plant breeding. Over the past 10 years confocal microscopy has become the tool of choice to obtain 3D and 4D information on the development of organ shape and the dynamics of gene expression patterns [1]. However, observations by confocal microscopy necessitate the presence of fluorescent dyes or proteins, generally achieved by plant transformation. It also requires a depth of the structure smaller than 50 µm for 3D reconstruction. The production of transgenic reporter lines by plant transformation remains very cumbersome or is simply not feasible in the majority of plant species and cannot be applied to hundreds of accessions that need to be phenotyped for quantitative genetics studies. Consequently the interest in alternative methods applicable to any type of non-transformed plant organ or tissue is ever increasing. Techniques such as magnetic resonance imaging, positon emission tomography, optical coherence tomography or X-ray absorption tomography have been used with various success depending on the size and physical properties of the biological material [2–7].

In this framework, X-ray absorption radiography or tomography is a widely used non-destructive method specially on dry seeds (see for instance [8–11]). The contrast in atomic number, at the physical origin of contrast in X-ray imaging, is strong in dry tissues because of the presence of air networks. However, in metabolically active “wet” tissues, in which the cell content consists essentially of water, the contrast in atomic number dramatically drops and consequently limits the domain of applicability of X-ray imaging for the characterization of fresh tissues. A possible way to enhance the contrast in X-ray imaging is to use contrast agents. This approach, very common in the biomedical domain, has only recently been introduced in plant sciences [12–14] and the selection of contrast agents and their penetration into wet plant tissues remains an open problem. An alternative way to enhance the contrast in X-ray imaging is to move from absorption tomography to in-line phase contrast tomography with coherent X-ray sources accessible via synchrotron radiation.

In X-ray in-line phase contrast imaging, the refraction of a partially coherent X-ray beam by an object of interest slightly modifies the original wave front profile. These variations result in changes in the locally transmitted intensity of the wave which contains quantitative information on the phase shift induced by the object. Due to its enhanced contrast over standard attenuation imaging, X-ray in-line phase contrast imaging is receiving more and more attention in medicine and biology (see for a recent review in medicine [15] and plant sciences [14, 16]). The enhanced contrast of X-ray in-line phase contrast tomography has so far been applied to various plant organs (see for the most recent review [14]), including the characterization of dry seeds [17–19] and developping wet tissues [20–22].

In previous work using X-ray phase contrast for imaging of developing wet plant tissues, the main focus was on the specific contrast due to local void spaces inside the plant tissues (seed [19, 22], fruit [21], leaf [20]) and their possible functional role on the physiology of these tissues or during ulterior imbibition. Distinctly, in this manuscript, the objective of phase contrast imaging is to exploit contrast between adjacent tissues to perform the global anatomical 3D segmentation of the compartments of a developing organism at various stages of development. The developmental stages of the seed considered in this manuscript precede the single mid-filling stage considered in the first publication which demonstrated the presence of the embryo in wet tissue of developing seeds [22]. We specifically focus on the early stages of seed development because they are characterized by major developmental events, during which some seed compartements grow while other disappear. These phenomenona are displayed in 3D after a detailed quantitative analysis of the recorded contrasts based on a segmentation process, whereas the previous demonstration of X-ray phase contrast imaging on seeds were illustrated with qualitative maximum intensity projections, or single thresholded 3D views colored for the eye with lookup tables. In this work, we demonstrate for the first time to our knowledge the value of X-ray in-line phase contrast imaging to developing seeds with a quantitative approach including the assessment of the observed phase contrasts and their use for segmentation purposes.

**Fig. 1:**
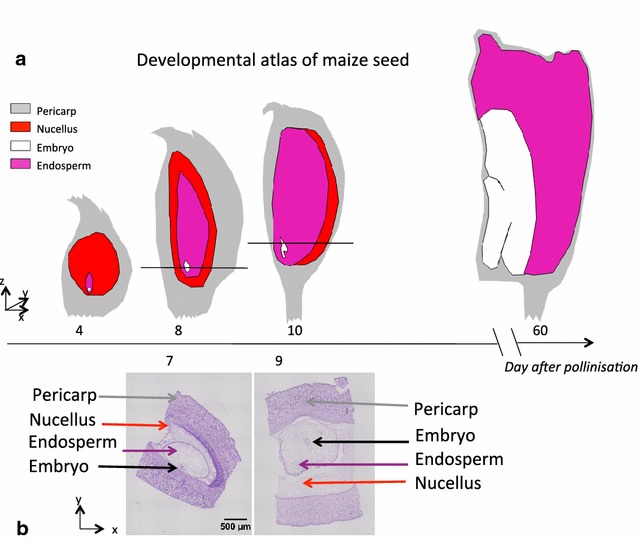
Developmental atlas of maize seed. **a** is a representation of maize seed development under the form of 2D drawings. **b** shows a conventional destructive histology pictures at 7 and 9 days after pollination (DAP). The level of the slice where these two pictures were extracted is indicated by the *black horizontal line* in the corresponding drawing in **a**

Among the major open challenges for imaging, figures seed development [23], which has been difficult to image because the seed is hidden from direct observation by the fruit case. It is also often rather small, for example only 0.7 mm in the model plant Arabidopsis. The focus on maize seed in the present work is based on its agronomic importance and its relatively large size of 12 mm at maturity. In maize, as in most species, the different compartments of the kernel enclose each other like Russian dolls. As visible in Fig. 1a, the embryo, which will give rise to the future plant, is surrounded by a nourishing tissue, the endosperm, which is embedded in the nucellus, itself enclosed by the pericarp. During kernel development the overall size of the kernel and the relative sizes of the four compartments undergo dramatic changes. The embryo and endosperm grow from single cells to structures of several mm in size, importing nutrients from the mother plant via the vascular system but also recycling metabolites released from the disappearing nucellus and pericarp. The dynamics of these changes differ between genotypes and influence the final size and composition of the kernel. To monitor these changes and to understand the aberrations occurring in mutant kernels, we set out to establish imaging techniques that allow to follow, for the first time in 3D, the development of embryo and endosperm inside the pericarp from 7 to 21 days after pollination (DAP). Here we demonstrate that this objective can be achieved with X-ray in-line phase contrast imaging at sufficient resolution to track the shape of the various subparts of the seed.

## Results

### X-ray absorption tomography

**Fig. 2:**
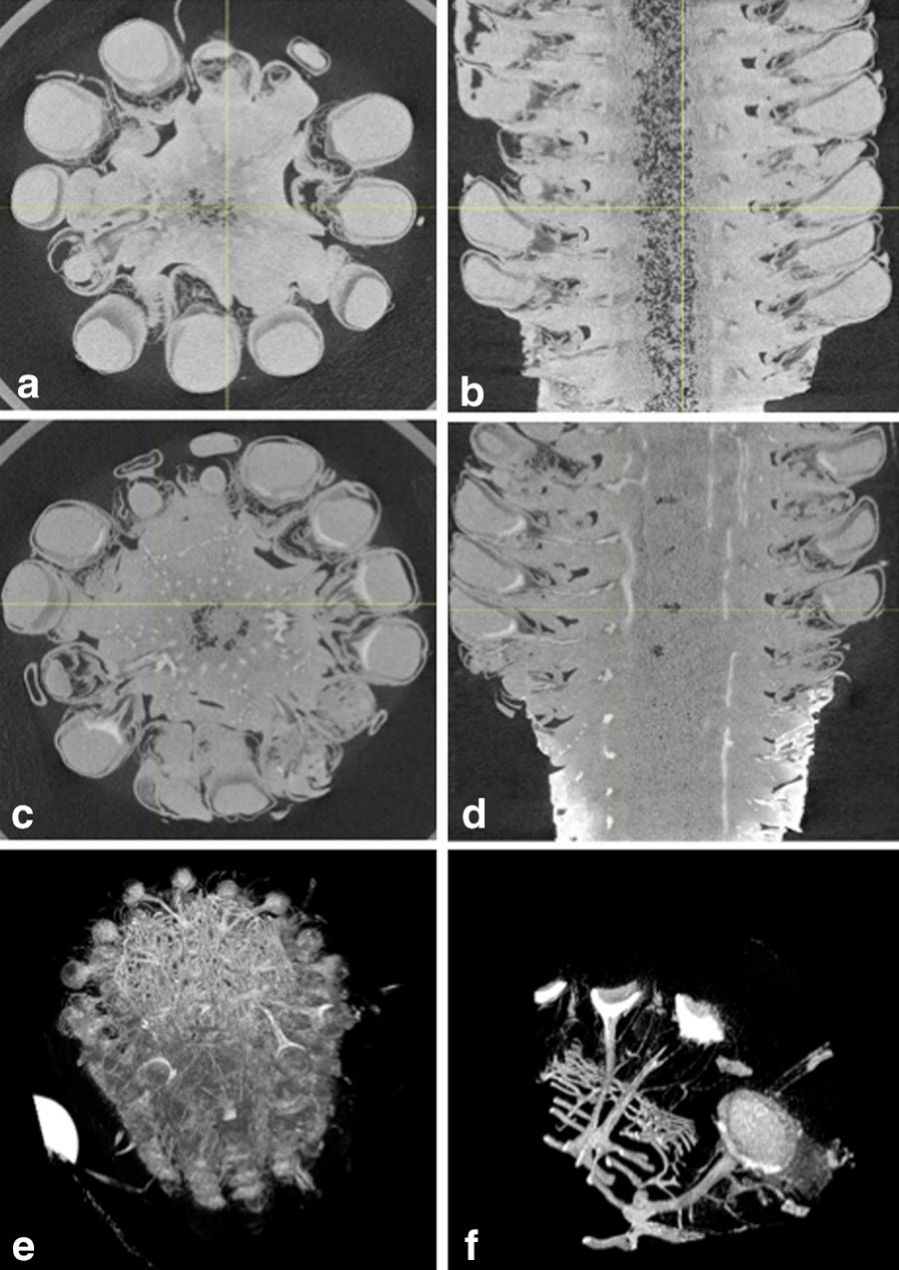
Maize seeds imaging by conventional absorption X-ray tomography without and with contrast agents. 12 DAP (Days After Pollination) old maize ear imaged by conventional absorption X-ray tomography and treated without contrast agents (**a** and **b**) or with Gadolinium (**c** and **d**) or Iobitridol (**e** and **f**) contrast agents during 24h prior to imaging. **a** and **c** represent transversal section of ear, whereas B and D represent longitudinal sections. 3-D reconstruction of the maize ear (**e**), and its zoom (**f**) in which only the brightest pixels are displayed, showing high X-ray contrast in ear vasculature and base of kernel

In a first instance conventional X-ray absorption tomography was carried out (see “Methods”) on developing maize seeds at 12 DAP. This was done at a spatial resolution with voxel size of 30 µm to give an overall view of the entire maize fruit. Even with such a low resolution, the contrast between the different kernel compartments appeared very weak between pericarp and endosperm, whereas it was absent between embryo and endosperm (Fig. 2a, b). Consequently, we were not able to perform robust image processing to extract these compartments. In subsequent experiments we tried to enhance contrast by the use of contrast agents. The base of the maize ear was placed in solutions used in perfusion computed X-ray absorption tomography for biomedical applications. It appeared in our tests that both solutions with Gadolinium and Iobitridol were able to irrigate almost the entire vascular network of the ear in less than 1 h (Fig. 2c–f), but that both contrast agents remained blocked at the level of the pedicel and would not enter the seed, even after several days.

### X-ray in-line phase contrast image acquisition and reconstruction

To visualise the overall morphology of embryo, endosperm, nucellus and pericarp by X-ray in-line phase contrast image different maize seeds were harvested at 7, 9, 12 and 21 days after pollination (DAP) and imaged individually. These stages were chosen because they cover the transition from morphogenesis (1 to 15 DAP) to seed filling (15 to 45 DAP) and include the nonmonotonic growth and disappearance of the nucellus together with the growth of the embryo and the endosperm from 0.5 mm/1.2 mm at 7 DAP up to 4/12 mm at 21 DAP (for a review see [23]).

All the seeds were imaged on the synchrotron radiation beamline ID19 of the European Synchrotron Radiation Facility (ESRF) with a beam of energy 17.6 keV and a detector positioned at 1 m from the sample in order to enhance propagation phase contrast. The 2D projections of a stack of 1000 virtual slices were acquired in 20 min. The pixel size was set to 5 µm by the optics of the detector. After applying Paganin’s algorithm for phase retrieval, the phase maps were used as input to a tomographic reconstruction algorithm and ring artefacts were removed.

Conventional histology was performed on different maize seeds taken at the same dates after pollination to correlate the size and shape of kernel compartments with zones of different contrast in X-ray in-line phase tomography (Fig. 1b). Despite cautious care, part of the tissues was retracted and/or damaged during the fixation, inclusion, sectioning and staining steps, as this is very often the case. This is why a 3D reconstruction with non-destructive 3D microscopy such as X-ray in-line tomography is expected to bring a specific added value by comparison with conventional histology.

### Phase contrast assessment

**Fig. 3:**
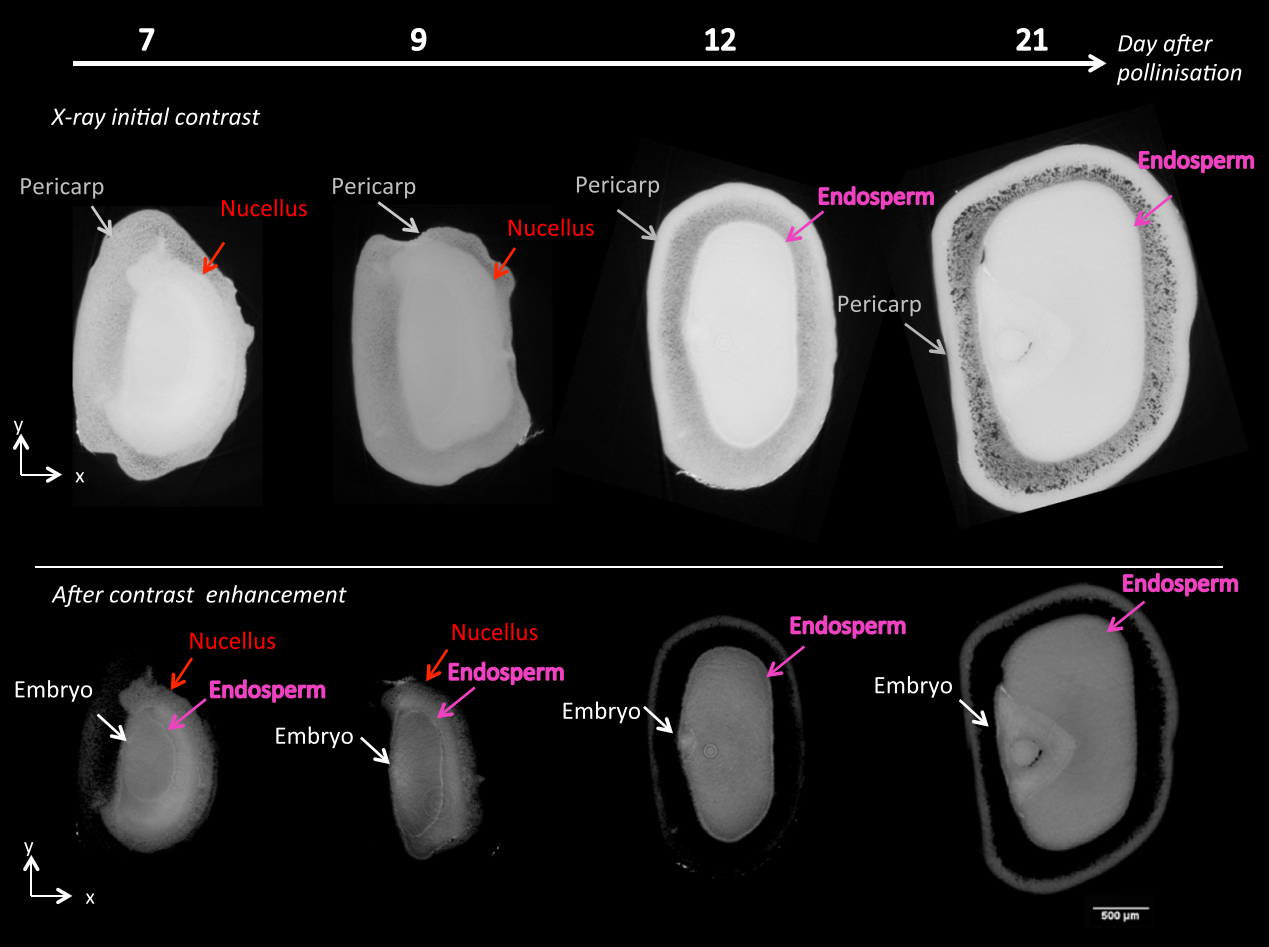
X-ray in-line phase tomography of maize seeds at 4 dates after pollination. *Top line* initial contrast enhancement on the whole *gray* level dynamic. *Bottom line* alternative contrast after dynamic reduction

Phase contrast images after reconstruction were coded on 32 bits. This made visualization of X-ray tomography difficult since the human eye is capable of differentiating approximately 200 gray levels. Contrast enhancement greatly helps the visual identification of the different compartments of the maize seed as illustrated in Fig. 3, which pictures two views of the same 2D slice with two contrast enhancement variants.

In most of the literature demonstrating the interest of X-ray for plant sciences, only such qualitative vizualization of contrast is given. To provide more quantitative insight on the quality of the images and to guide the subsequent segmentation strategy, the contrasts between the different seed compartments were computed with the Fisher ratio in Table 1. The higher the value of this contrast index, the easier the segmentation of the frontier between two compartments. As visible in Table 1, the difficulty of this segmentation task varies. Consequently, different strategies were implemented for the segmentation of each compartment of the maize seed.

**Table 1:** Contrast measured by the Fisher ratio $F_r$ between compartment of the maize seed with X-ray in-line phase tomography at 7, 9, 12, and 21 DAP

Compartment	$F_r$ at 7 DAP	$F_r$ at 9 DAP	$F_r$ at 12 DAP	$F_r$ at 21 DAP
Pericarp-nucellus	14.0	11.9	NA	NA
Nucellus-endosperm	2.2	2.2	NA	NA
Pericarp-endosperm	NA	NA	28.9	12.8
Albumen-embryo	0.9	0.8	0.2	2.2

*NA* not applicable

### Image segmentation

**Fig. 4:**
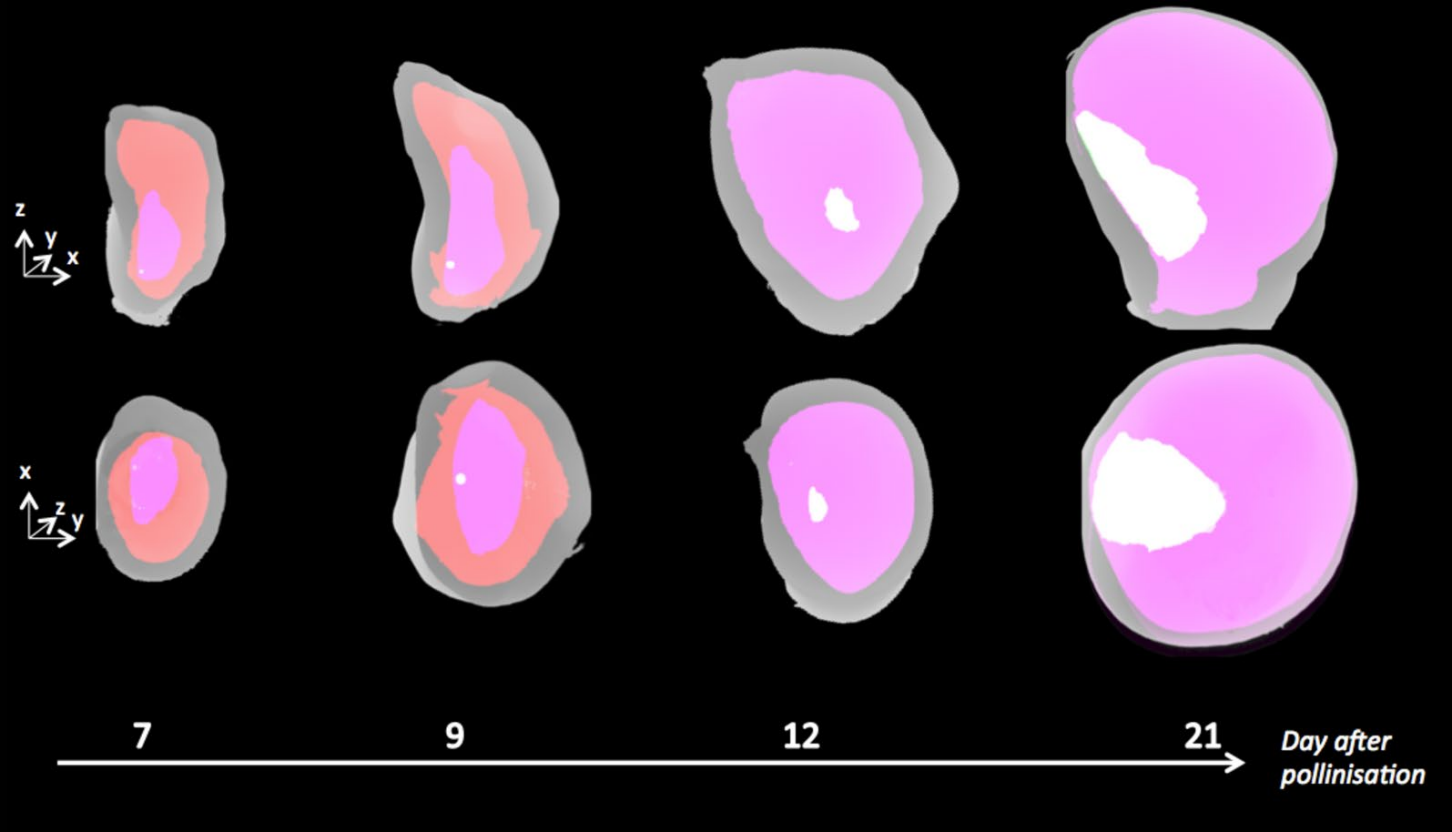
3D segmentation of maize seeds at 4 different developemental stages corresponding to 7, 9, 12 and 21 days after pollination. The segmentation represented under two different angles of view are to be compared with the usual 2D respresentation in Fig. 1. The *colors* correspond to* grey* (pericarp),* red* (nucellus),* pink* (endosperm),* white* (embryo). A video of these segmentations is available Additional file 1.

The pericarp and nucellus, which naturally present high contrast at all stages, were segmented by simple thresholding. The endosperm compartment was found not sufficiently contrasted to be segmented by a simple thresholding method. Instead, we took advantage of the presence of edges at the frontier between endosperm and nucellus, together with the prior information that endosperm is expected to be a closed contour, and successfully implemented an active contour method. Concerning the embryo, most subtle in contrast as shown in Table 1, we performed manual segmentation at 7, 9 and 12 DAP, whereas an active contour was possible at 21 DAP. The 3D segmentation of the maize seed at the four developmental stages is given in Fig. 4 under two angles of visualization. This is completed by a video attached as Additional file 1 and downloadable on the website of the journal. This demonstrates the capability of X-ray in-line phase tomography coupled to our image reconstruction and processing pipeline to capture the nonmonotonic growth and disappearance of the nucellus together with the growth of the endosperm and the embryo.

**Fig. 5:**
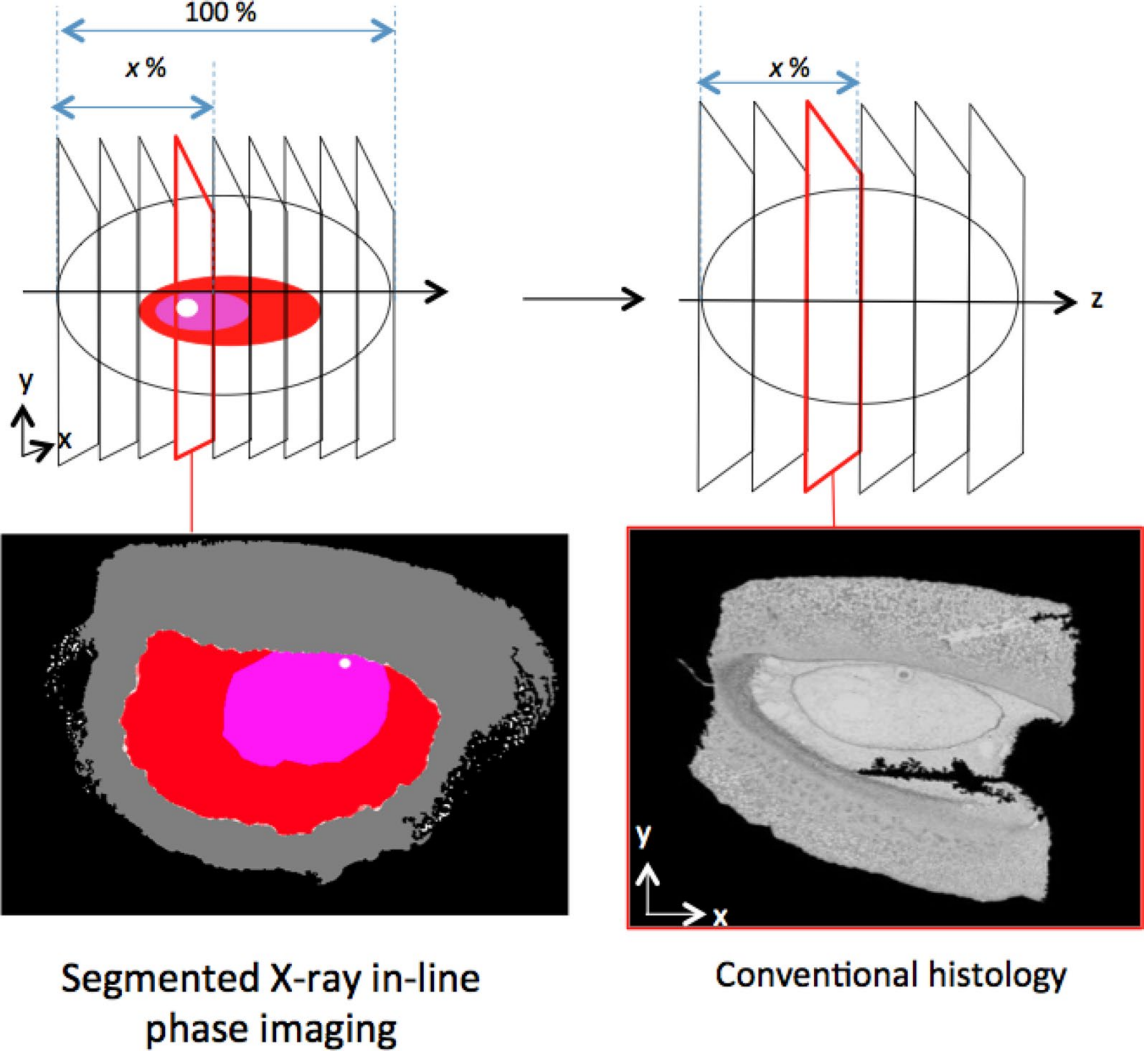
Comparison of a virtual slice obtained from X-ray in-line phase tomography with a real slice obtained from conventional histology. As illustrated in* upper panel*, slices were taken at the same location in maize seeds both taken at 7 DAP. For X-ray in-line phase image, the* colors* correspond to pericarp (*grey*), nucellus (*red*), endosperm (*pink*) and embryo (*white*). The length of the pericarp along the X-axis is taken as the reference (100 %) and the x % correspond to the position of the slice

In order to validate our virtual histology made by X-ray in-line phase tomography, we compared it with conventional histology. Conventional histology was done by imaging serial sections of seed and then positioning these images in their respective order of apparition to constitute a stack of the seed (see “Methods” section and Fig. 5). The comparison of the X-ray in-line phase tomography data with those obtained by conventional histology for a seed at 7 DAP demonstrated a good match for the estimation of the length of the different compartments with both methods (Table 2). As a complementary element of comparison Fig. 5 depicts the respective slice taken at the location corresponding to the beginning of the embryo. It appears that they correspond with a bias of only 1 % to the same location in the stack images. This is a very good agreement if one considers that this was obtained with two distinct seeds of the same age.

**Table 2:** Comparison of global measures of length between X-ray in-line phase tomography and conventional histology of different maize seed compartment at 7 DAP

Imaging technique	X-ray in-line phase tomography (%)	Conventional histology (%)
Nucellus	85	85
Endosperm	47.5	52
Embryon	1.2	2.0

The reference 100 % is taken as the length of the pericarp

## Conclusion

In this article we demonstrate the feasibility of 3D imaging of maize embryo and endosperm morphology inside the seed coat with X-ray imaging during early developmental stages when both are metabolically highly active and the cells essentially filled with water. The contrasts are too poor for segmentation in conventional absorption X-ray but large enough with X-ray in-line phase contrast imaging. The coherent beam of X-ray allows to visualize and segment the four main compartments of the seed during morphogenesis without having to resort to the use of contrast agents. The cumulated data from four different developmental stages establish for the first time a 3D dynamic picture of the growth of embryo and endosperm and the concomitant reduction and nutritional recycling of the seed coat (nucellus and pericarp). The sizes and shapes of the compartments obtained by non-destructive 3D virtual histology were compared with success to knowledge from the literature [23] and data obtained in parallel by conventional destructive histology. The acquisition time was 20 min for a single seed with X-ray in-line phase contrast imaging. This is much shorter than the time requested for the realization of fixation, inclusion, sectioning and scanning of a single seed with destructive histology. X-ray in-line phase contrast imaging can thus be considered as fast by comparison with destructive histology which is the most current reference for anatomical atlas in plant biology. Comparison between histology and X-ray phase contrast imaging was also proposed in [22]. This was at the cellular scale to demonstrate that the phase contrast was correlated with the presence of gaz in the tissue. In this work, thanks to the use of automated histological scanning system, we performed a global comparison at the scale of the entire seed which shows good agreement on the size of the measured compartments in the seed by the destructive histological technique and non destructive 3D imaging by X-ray phase contrast tomography. For this successful outcome a complete image processing pipeline had to be developed, which described how to take benefit of the contrast between the various subparts of the maize seed. We identified and quantitatively assessed the level of difficulty for the segmentation of the different compartments of the seed, the most challenging being the segmentation of the embryo before 12 DAP. Such challenges are now open for studies with more advanced image processing tools.

The use of X-ray in-line phase tomography, not only promises to outperform conventional histology in terms of time of acquisition and precision for 3D measurement on native samples. It also enables, thanks to its high sensitivity to interfaces, to highlight specific areas that did not attract particular attention in conventional histology and are not referenced in atlases of developmental biology. This is for instance the case, with the very textured part located after the outer part of the nucellus at DAP 21 visible in Fig. 3. Thanks to the quantitative comparison with conventional histology, we are in position to state that this textured region is part of the pericarp. The texture can physically originate from multiscale interfaces, which would be compatible with a porous air-tissues interface. This conjecture is now open for further investigations.

This proof of concept for quantitative use of X-ray in-line phase contrast for developing seeds also pinpointed several limitations of this method. The most obvious one is the lack of cellular resolution when compared to conventional histology. This could possibly be overcome by higher spatial resolution imaging a small part of the sample in a local tomography mode inside the same type of seeds. In addition, the obtained results were established in non-natural conditions since for room constrains, the grains were extracted from their ear during the 20 min acquisition. Also, because of the limitation of the temporal access to the synchrotron beam, it was not possible to realize a time lapse experiment monitoring the morphogenesis of a single seed throughout different developmental stages. Consequently, the knowledge of the possible impact of the X-ray dose on maize seed development was not accessible. New development in coherent X-ray sources accessible to standard lab environments may open access to such complementary experiments. Desktop phase contrast micro-CT devices are beginning to develop. The usual solution is to use interferometry which requires several scans of the sample with adapted gratings yielding to long scan times, and is generally limited to a spatial resolution of a few micrometers, thus much higher than what was used here. The other possibility is to exploit edge enhancement effect with microfocus sources but such systems are known to offer less monochromaticity and spatial coherence than the synchrotron facility. Consequently, the assumption required for the validity of the phase reconstruction algorithm together with the assumption required for the tomographic reconstruction are not satisfied. This contrast is thus expected to be of lower quality with a desktop phase contrast micro-CT than with a synchrotron facility. To our knowledge phase CT on a desktop system has so far never been demonstrated. Although not yet accessible in routine, synchrotron radiations are now available worldwide as national or transational public facilities with an acquisition time of 20 min enabling to realize the 3D segmentation of some 72 seeds during a typical 24H run. Such acquisition campaigns could now be undertaken thanks to the methodological details on image acquisition and post-processing provided in our article for fast 3D virtual histology compared to the conventional destructive 2D histology.

The domain of 3D imaging techniques with cellular resolution and field of view compatible with the smallest entire plant organisms, i.e. seed or seedling, is progressing very rapidly [8]. It would be interesting to develop a similar approach to the one developed in this article for developmental imaging with other imaging techniques. Optical coherence tomography, another 3D virtual histology, was for instance unfruitful for maize seed screening because of too large thickness of the tissues while it is very adapted to the screening of the development of seedlings of Arabidopsis as demonstrated in [8]. This illustrates that the knowledge of which 3D microscopy technique is suited for which biological question is open for further investigations such as the one presented in this article for X-ray in-line phase contrast imaging applied to maize seed morphogenesis.

## Methods

### Plant material

Maize plants of genotype A188 were grown in a greenhouse with a 16 h illumination period (100 $\rm W/m^2$) at 24/19 °C (day/night) and without control of the relative humidity. At maturity the plants underwent controlled self pollination. The substrate, watering and fertilizing were as described previously [24].

### Histology

Maize seed sides were cut for better penetration of the fixative and seeds fixed with paraformaldehyde 48 h at 4 °C, dehydrated in an ethanol series and included in paraplast with a Leica TP1020 benchtop tissue processor. 10 µm thick serial sections were obtained with a Leica RM2235 rotary manual microtome. Histoclear was used to remove the wax. After rehydration in a decreasing ethanol series the tissues were stained by periodic acid and Schiff’s reagent (Sigma). Automated acquisitions were realized on a slide scan Axioscan Z1 (Zeiss). It took approximately 8 h to acquire the all set of serial sections representing an entire seed. The slices were automatically positioned in their respective order of apparition to constitute a stack of the seed.

### X-ray absorption tomography

Self pollinated ears were separated from the plant and soaked up to 48 h in either Gadoteric acid dye (Dotarem) at 0.5 nmol/ml or Iobitribol dye (Xenetix) at 300 nmol/ml. Ear sections containing 5 rows of kernels were imaged on a GE Phoenix v—tome—x equiped with a nanofocus X-ray tube and a Varian paxscan detector. The tomograph is described in more details in [25]. It was operated with a 80kV acceleration voltage using a tungsten transmission target with a 280 µm current. The number of projections was 900, each radiograph was an average of 3 exposures of 333 ms each to reduce the noise. The voxel size was set at 30 µm. The acquisition time was 20 min.

### X-ray in-line phase contrast image acquisition and reconstruction

For X-ray in-line phase tomography, raw projection of the seeds were acquired on the synchrotron radiation beamline ID19 of the European Synchrotron Radiation Facility (ESRF) in Grenoble, France, with an experimental image acquisition characterized by a beam of energy 17.6 keV, selected from undulator radiation using Al filters. The X-ray beam transmitted through the specimen is acquired on a detector using a LuAg scintillator screen, visible light optics, and a 2048 × 2048 CCD detector with 32 bit resolution. The detector was positioned at 1 m from the sample for in-line phase-contrast imaging. The acquisition duration was 20 min per seed. Phase retrieval was performed from a single phase-contrast image at each projection angle, using Paganin’s method [26] where a low-pass filter followed by a logarithmic operator is applied. The term ‘in-line’ in this context of X-ray phase contrast tomography is a term used in physics because the method used to capture the phase information requires only a single projection by angle while other physical methods based on gratings, or interference demand more acquisitions. The so-called $\delta /\beta$ coefficient which governs the cut-off frequency of this filter was chosen at $\delta /\beta = 300$ following the approach given in [27]. After phase retrieval, the phase maps were used as input to a 3D parallel-beam tomographic reconstruction algorithm based on the filtered back projection algorithm described in [28]. The resulting 3D volume is a stack of 1000 slices of 2048 × 2048 voxels and slice thickness equal to pixel size (isotropic voxels) 5 µm. For each scan, this procedure provided a reconstructed 3D volume which suffers from spatially circular noise typical of reconstruction artefacts in tomography caused by differences in the individual pixel responses of the detector. These ring artefacts were removed with the nonlinear median-based filter algorithm of [29].

### Contrast metric

The contrasts between each compartment of the seed were computed with the Fisher ratio *F*_r_ given by:

1 $$\begin{aligned} F_{r}=\frac{( \mu _1-\mu _2)^2}{\sigma _1^2+\sigma _2^2} \, , \end{aligned}$$

which takes as input the average gray level values $\mu _1$ and $\mu _2$ of two compartments of the seed and their respective variances $\sigma _1^2$ and $\sigma _2^2$.

### Image segmentation

**Fig. 6:**
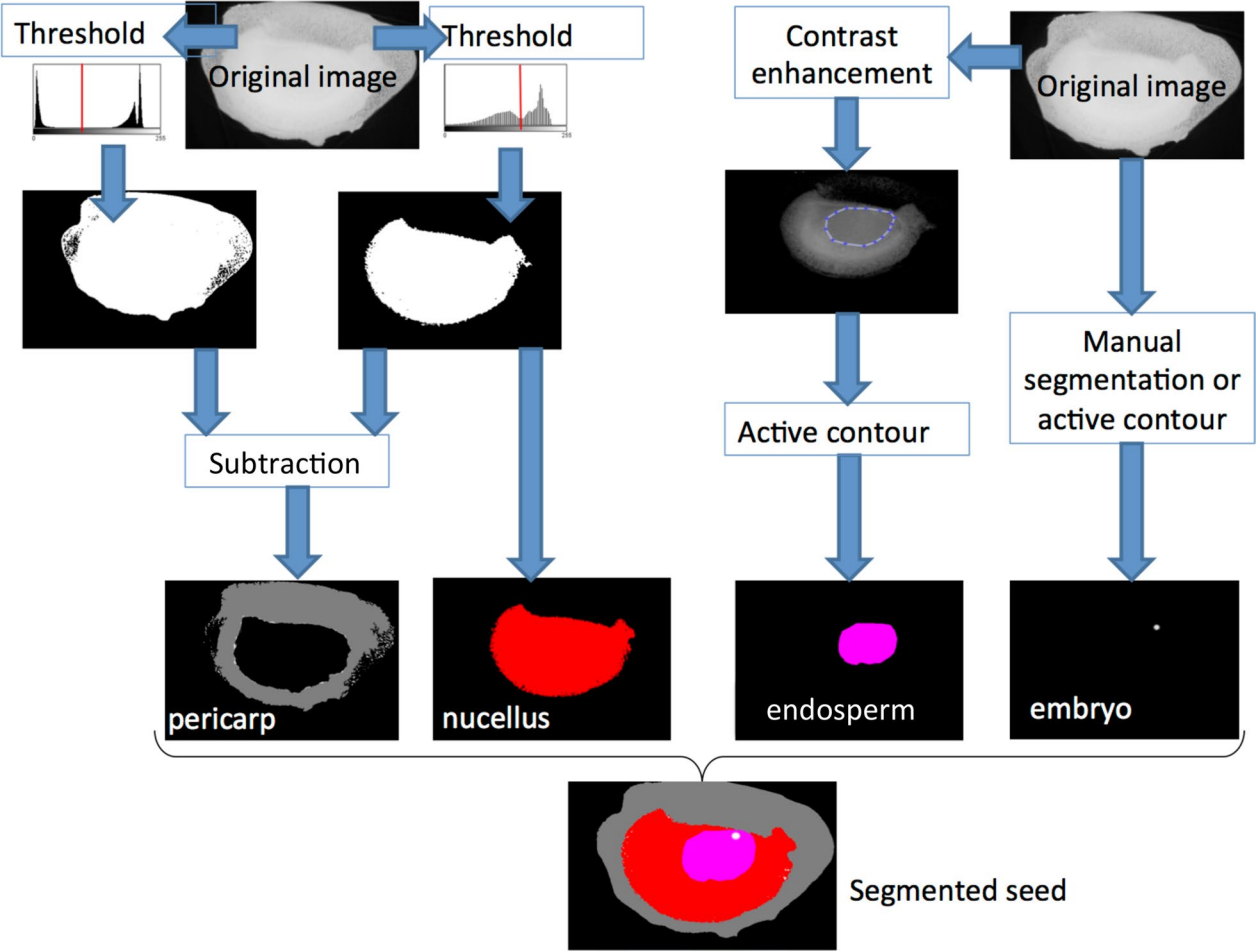
Image processing pipeline developed for the segmentation of the different compartements of maize seed with X-ray in-line phase tomography

The global image processing pipeline developed for the segmentation of each compartment of the maize seed is given in Fig. 6. The pericarp and nucellus were segmented by simple thresholding. For the endosperm an active contour method based on the level set method [30] was therefore used with the Matlab implementation of the freely available version of [31]. The following parameters $number\_of\_iteration= 50$, $propagation\_strengh= 0.2$, $threshold = 1$ were found to be robust for the segmentation of the endosperm at the four stages considered. The active contour was initiated with a manually positioned bounding box including the endosperm on a 2D slice of the X-ray stack of images. After convergence of this manual initiation step to the segmentation of the endosperm on this specific slice, the same active contour algorithm was applied on the consecutive slice. The initial bounding box for the active contour of this new slice was taken as the result of the extracted contour of the previous slice. This process was iterated so as to process the whole stack. The first slice was taken in the middle of the seed so as to parallelize the process in both possible directions from this slice. Concerning the embryo we performed manual segmentation at 7, 9 and 12 DAP and used active contour at 21 DAP.

## Additional file

10.1186/s13007-015-0098-y 3D segmentation of the maize seed at the four developmental stages.
